# The association between length of stay in the emergency department and short-term mortality

**DOI:** 10.1007/s11739-021-02783-z

**Published:** 2021-06-10

**Authors:** Torgny Wessman, Johan Ärnlöv, Axel Carl Carlsson, Ulf Ekelund, Per Wändell, Olle Melander, Toralph Ruge

**Affiliations:** 1 Department of Clinical Sciences, Faculty of Medicine, Lund University, Malmö, Sweden; 2 Emergency Department, Skåne University Hospital, Malmö, Sweden; 3 Division for Family Medicine and Primary Care, Department of Neurobiology, Care Sciences and Society, Karolinska Institutet, Huddinge, Sweden; 4 School of Health and Social Studies, Dalarna University, Falun, Sweden; 5 Department of Clinical Sciences Lund, Emergency Medicine, Faculty of Medicine, Lund University, Lund, Sweden; 6 Academic Primary Health Care Centre, Region Stockholm, Stockholm, Sweden

**Keywords:** Mortality rate, Emergency room, Emergency department length of stay, Emergency medicine, Emergency department crowding, Epidemiology, Elderly

## Abstract

The detrimental effects of increased length of stay at the emergency department (ED-LOS) for patient outcome have been sparsely studied in the Swedish setting. Our aim was to further explore the association between ED-LOS and short-term mortality in patients admitted to the EDs of two large University hospitals in Sweden. All adult patients (> 18 years) visiting the ED at the Karolinska University Hospital, Sweden, from 1/1/2010 to 1/1/2015 (*n* = 639,385) were retrospectively included. Logistic regression analysis was used to determine association between ED-LOS and 7- and 30-day mortality rates. All patients were triaged according to the RETTS-A into different levels of medical urgency and subsequently separated into five quintiles of ED-LOS. Mortality rate was highest in highest triage priority level (7-day mortality 5.24%, and 30-day mortality 9.44%), and decreased by lower triage priority group. For patients with triage priority levels 2–4, prolonged ED-LOS was associated with increased mortality, especially for lowest priority level, OR for priority level 4 and highest quintile of ED-LOS 30-day mortality 1.49 (CI 95% 1.20–1.85). For patients with highest triage priority level the opposite was at hand, with decreasing mortality risk with increasing quintile of ED-LOS for 7-day mortality, and lower mortality for the two highest quintile of ED-LOS for 30-day mortality. In patients not admitted to in-hospital care higher ED-LOS was associated with higher mortality. Our data suggest that increased ED-LOS could be associated with slightly increased short-term mortality in patients with lower clinical urgency and dismissed from the ED.

## Introduction

Emergency department (ED) crowding is a term that is used to describe a phenomenon in the ED in which there is an imbalance between needs and access to proper care [1]. ED crowding has been related to treatment delays, medical errors, and increased patient morbidity and mortality [2, 3]. ED crowding seems to be an international problem, although it has been described differently in separate countries [4].

ED length of stay (ED-LOS) is a well-accepted ED performance indicator and is closely related to crowding, quality of care and patient outcome [5]. Several studies, including trauma and non-trauma patients as well as patients with high and low clinical priority, have shown an association between increased ED-LOS and unfavourable outcomes such as increased mortality and in-hospital length of stay [6, 7].

The main aim of ED triage is not to predict short-term mortality but to early identify patients with high acuity. High acuity patients will be seen first, and triage priority is therefore closely associated with ED-LOS. In addition, ED-LOS depends on the patient-specific care, including blood sampling and diagnostic imaging, admission to in-hospital care or not, and the hospital bed occupancy. The patient’s presenting symptoms as well as the presence of senior or junior doctors may also affect ED-LOS [8, 9].

The association between ED-LOS and patient mortality has been sparsely studied in the Swedish ED setting. A recent study has shown an association between increased ED-LOS and increased mortality in patients with low medical urgency and patients not admitted to in-hospital care [10]. These data have, however, not been confirmed by others and our aim was therefore to further explore the association between ED-LOS and short-term mortality rate in patients admitted to two of Sweden largest University hospital emergency departments.

## Material and methods

### Study design

This was a retrospective cohort study in ED patients.

### Study setting and population

The locations were two university hospitals of Stockholm, Karolinska University Hospital Huddinge and Solna. The ED is open for adult people 18 years of age and above with somatic diseases (internal medicine, surgical, orthopaedic, neurologic and infectious conditions), and is run by emergency-trained physicians. At ED Total (Huddinge and Solna) annual ED visits were around 150,000 patients. Between 1/1/2010 and 31/12/2014, 641,314 visits at the two hospitals ED’s were included in this study and analysed. The patient data were extracted from the hospital administrative system. Patient data were excluded if there was no full documentation of all variables, if patients died upon arrival to the ED or if the patient had an ED LOS > 4000 min (values above 4000 min were assumed not to be probable and interpreted as typing errors) leaving 639,385 patients that were included in the present analyses.

### Study protocol

At arrival to the ED at both hospitals, the patient visit is immediately registered and triage according to RETTS-A is performed by nurses [11]. RETTS-A Triage priority is determined using a combination of the patient’s presenting symptoms and signs in addition to vital sign values. The RETTS-A triage scale priorities are: red, orange, yellow, green, and blue, in declining priority of acuity, i.e. triage priority 1–5. The two highest levels of acuity (red and orange) represent potential life-threatening conditions; whereas, the levels 3–4 (yellow, green) represent stable patients in need of acute care. Level 5, i.e. blue priority, representing non-urgent complaints. The patient’s presenting symptoms are matched to one of 59 Emergency Symptoms and Signs (ESS) algorithms in accordance with RETTS-A. The vital signs for each triage level have specific cut-off values indicating different levels of acuity. The chief complaint algorithms are known as emergency symptoms and signs for emergency care. Each emergency symptom and sign includes one or more chief complaints and is classified according to the International Statistical Classification of Diseases and Related Health Problems, 10th Revision, 2007 (ICD-10), and a RETTS-A logistic process is attached to each algorithm.The more urgent of either the vital signs or presenting symptoms and signs becomes the patient’s final triage priority. Patients with blue priority, representing non-urgent complaints and minor injuries, were referred to a primary acute health care centre from the ED and therefore not included in this study. Patients with green priority have vital signs in, or close to, normal range and thus less urgent complaints than yellow, orange, and red patients.

### Data collection and variables

The collected variables were age, sex, any of the ten most common chief complaints pre-defined by RETTS-A (abdominal pain, chest pain, shortness of breath, painful or swollen extremity, malaise, dysrhythmia, allergic reaction, syncope, intoxication, fever and undefined), triage priority at arrival, if the patient was given prehospital care given by ambulance or not, if the patients were admitted to in-hospital care or not, if the patient presented to the ED in the weekend or not. The chief complaints can be seen as a crude proxy for comorbidity and should eliminate some confounding associated with complaint.

### Outcomes

Primary outcomes were 7- and 30-days mortality, counted from registration to the ED. Information on patient survival as dependent variable was extracted from the Swedish population register, administrated by the Swedish Tax Agency, which includes every Swedish resident and has a high validity and completeness. Thus, there was a near complete follow-up of 7- and 30-days mortality for every patient visiting the EDs included in this study.

### Statistical analyses

We present descriptive data on the study cohort including mean and standard deviation for baseline characteristics. Patients were categorised into different quintiles of ED-LOS to provide further insights into potential threshold effects on these associations. Pearson *χ*^2^ test and One-way ANOVA was used for comparison across groups. Multiple logistic regression models were performed to investigate the relationship between ED-LOS, continuous model and quintile model, and mortality. The model included age, sex and triage-priority, the ten most common chief complaints, prehospital care given or not, in-hospital care, when the patient presented to the ED (day of the week and time of the day) and the diagnosis at the ED. We tested multiplicative interactions and stratified instead of adjusting when needed. Odds ratios (OR) and 95% confidence intervals (CI) are presented. *p* values < 0.05, two-sided, were considered significant. Statistical analyses were performed using the software STATA version 13.

A permit was issued from the Ethics Review Board in Stockholm, reference number: 2017/1252-31/1.

## Results

### Baseline characteristics

Different patient categories and ED-LOS are described in Table 1. The age distribution was as follows, patients 18–59 years represented 58% of all patients, patients between 60 and 79 years represented 27% and patients’ ≥ 80 years represented 15% of the total number of patients. Patients’ age ≥ 80 had the longest ED-LOS, 297 min, patients between 60 and 79 years had an ED-LOS of 266 min and patients between 18 and 59 years had an ED-LOS of 235 min. Most patients (70%) were not admitted to in-hospital care. Non-admitted patients had shorter visiting time, 237 min compared to 260 min for admitted patients (*p* < 0.001). The longest ED-LOS was observed for patients triaged with priority 3 (*p* < 0.001).

**Table 1:** Patient categories and emergency department length of stay (ED-LOS)

Categories	ED-LOS time (mean, min ± SD, min–max)	*n*
Sex		
Men	252 ± 163	289,882
Women	253 ± 164*	349,503
Age		
18–59	235 ± 152	373,231
60–79	266 ± 170*	176,960
≥ 80	297 ± 181*	91,123
In-hospital care		
Admitted patients	260 ± 195*	190,477
Non-admitted patients	237 ± 146	450,837
Triage		
Triage priority 1	214 ± 160 (1–2370)	34,363
Triage priority 2	260 ± 162 (8–2783)*	96,913
Triage priority 3	276 ± 173 (0–3977)*	265,826
Triage priority 4	229 ± 150 (2–3645)*	244,212
Quintiles		
Quintile 1	86 ± 27 (0–124)	123,098
Quintile 2	155 ± 17 (125–184)	129,216
Quintile 3	216 ± 19 (185–251)	129,082
Quintile 4	296 ± 28 (252–350)	129,264
Quintile 5	498 ± 174 (351–3977)	130,654

*ED-LOS* Emergency department length of stay

*Indicates statistically significant difference, *p* value < 0.05

The sample divided by quintiles of ED-LOS is shown in Table 2. The mean age increased by increasing ED-LOS, as did rate of in-hospital care, while risk of intensive-care decreased. The rate of patients in triage priority 3 was highest in the highest quintile, while the rate of patients in triage priority 4 (lowest priority urgency) was highest in the lowest quintile of ED-LOS.

**Table 2:** Characteristics of patients by quintiles of emergency department length of stay (ED-LOS)

	Quintile 1 86 ± 27 min	Quintile 2 155 ± 17 min	Quintile 3 216 ± 19 min	Quintile 4 276 ± 173 min	Quintile 5 498 ± 174 min
Age (years, mean ± SD)*	49 ± 0.06	52 ± 0.06	54 ± 0.06	56 ± 0.06	59 ± 0.06
Sex (% female per quintile)*	55	54	54	54	55
In-hospital care (% per quintile)*	23	25	29	31	39
Triage priority (% per quintile)					
Priority 1	9	5	5	4	4
Priority 2	11	16	17	16	15
Priority 3	30	39	42	45	50
Priority 4	50	40	36	35	31
Intensive care within 72 h (% per quintile)*	1.25	0.60	0.45	0.41	0.36
Mortality					
Mortality 7d (% per quintile)*	0.82	0.57	0.63	0.66	0.79
Mortality 30d (% per quintile)*	1.60	1.48	1.87	2.10	2.60

*Indicates statistically significant difference, *p* value < 0.05 (Pearson *χ*^2^-test)

### Mortality

The number of deceased patients per quintile of ED-LOS expressed as percent are shown in Table 2. The number of deceased patients within 7-days showed highest rates in the 1st and 5th quintile, 0.82% and 0.79%, respectively, and with lower rate of deceased patients in the middle three quintiles. The. In contrast, the number of patients deceased within 30-days peaked in the 5th quintile, 2.60%, compared to 1.60% in the 1st quintile, with otherwise increasing death rates from the 2nd to the 5th quintile. The rate of deceased decreased from triage priority 1 to triage priority 4 (Table 3).

**Table 3:** Mortality per triage priority expressed as percent deceased patients per triage priority

	Triage priority 1	Triage priority 2	Triage priority 3	Triage priority 4
Mortality				
Mortality 7d	5.24	1.28	0.44	0.09
Mortality 30d	9.44	3.65	1.64	0.47

Data are expressed as % per ED-LOS quintile

In patients with triage priority 1 (high medical urgency), a negative association between ED-LOS and both 7- and 30-day mortality was observed. This association seemed to be more pronounced with increasing ED-LOS, i.e. and in ED-LOS quintile 5 compared to the 1st quintile for 7-day mortality OR 0.63 (CI 95% 0.53–0.74), and 30-day mortality OR 0.84 (CI % 0.75–0.96), respectively (Table 4). In contrast, the opposite pattern appeared evident in triage group 3 and 4, here a positive association was observed between ED-LOS and mortality (Table 5).

**Table 4:** Logistic regression for the association between priority of triage, quintiles of emergency department length of stay (ED-LOS) and 7- and 30-day mortality

	Triage priority 1 OR (95% CI)	Triage priority 2 OR (95% CI)	Triage priority 3 OR (95% CI)	Triage priority 4 OR (95% CI)
7-day mortality				
Continuous model				
ED-LOS (hours)	**0.94 (0.92–0.96)**	0.98 (0.96–1.01)	**1.02 (1.00–1.04)**	1.03 (0.99–1.07)
ED-LOS quintile model				
Quintile 1 86 ± 27 min	referent	referent	referent	referent
Quintile 2 155 ± 17 min	**0.80 (0.70–0.92)**	0.94 (0.75–1.17)	1.15 (0.87–1.51)	1.04 (0.62–1.75)
Quintile 3 216 ± 19 min	**0.73 (0.63–0.85)**	1.13 (0.91–1.39)	1.24 (0.96–1.62)	0.79 (0.47–1.35)
Quintile 4 276 ± 173 min	**0.67 (0.57–0.78)**	0.97 (0.79–1.20)	**1.35 (1.05–1.74)**	1.03 (0.64–1.68)
Quintile 5 498 ± 174 min	**0.63 (0.53–0.74)**	1.00 (0.81–1.24)	**1.36 (1.06–1.74)**	1.25 (0.79–1.97)
30-day mortality				
Continuous model				
ED-LOS (hours)	**0.97 (0.96–0.99)**	**1.01 (1.00–1.02)**	**1.02 (1.01–1.03)**	**1.03 (1.01–1.05)**
ED-LOS quintile model				
Quintile 1 86 ± 27 min	referent	referent	referent	referent
Quintile 2 155 ± 17 min	0.93 (0.84–1.04)	1.00 (0.87–1.14)	**1.18 (1.02–1.37)**	1.04 (0.82–1.34)
Quintile 3 216 ± 19 min	0.92 (0.82–1.03)	**1.19 (1.04–1.36)**	**1.35 (1.18–1.55)**	**1.31 (1.04–1.65)**
Quintile 4 276 ± 173 min	**0.83 (0.73–0.93)**	1.13 (0.99–1.29)	**1.34 (1.17–1.53)**	**1.27 (1.02–1.60)**
Quintile 5 498 ± 174 min	**0.84 (0.75–0.96**)	**1.19 (1.04–1.35)**	**1.44 (1.26–1.63)**	**1.49 (1.20–1.85)**

Logistic regression, analyses adjusted for: age, sex, the ten most common chief complaints (to account for different risks associated with different complaints), prehospital care given or not, admission to in-hospital care, when the patient presented to the ED (day of the week and time of the day) and patient’s diagnosis. Quintile 1 = ref. Bold values are statistically significant

**Table 5:** Logistic regression for the association between ED-LOS (hours, continuous model) and 7- and 30-day mortality.

	Triage priority 1 OR (95% CI)	Triage priority 2–4 OR (95% CI)
7-day mortality		
Admission to in-hospital care	**0.92 (0.90–0.94)**	0.98 (0.97–1.00)
No admission to in-hospital care	**1.11 (1.06–1.15)**	**1.11 (1.09–1.14)**
Age 18–59 years	**0.90 (0.84–0.97)**	1.02 (0.98–1.07)
Age 60–79 years	**0.94 (0.91–0.97)**	**1.02 (1.00–1.04)**
Age ≥ 80 years	**0.92 (0.89–0.95)**	**0.97 (0.98–1.00)**
30-day mortality		
Admission to in-hospital care	**0.96 (0.95–0.98)**	**0.99 (0.99–1.00)**
No admission to in-hospital care	**1.10 (1.06–1.14)**	**1.12 (1.11–1.13)**
Age 18–59 years	**0.94 (0.89–0.98)**	**1.04 (1.01–1.06)**
Age 60–79 years	**0.96 (0.94–0.98)**	**1.03 (1.02–1.04)**
Age ≥ 80 years	**0.96 (0.94–0.98)**	**1.02 (1.00–1.02)**

Analyses were stratified by age, whether the patient was admitted to in-hospital care or not and triage priority. Logistic regression, analyses adjusted for: sex, the ten most common chief complaints (to account for different risks associated with different complaints), prehospital care given or not, in-hospital care, when the patient presented to the ED (day of the week and time of the day) and patient’s diagnosis. Bold values are statistically significant

We observed an interaction between triage priority, as well as hospital care, and most other variables. As a result, we decided to stratify analyses by age-group, triage priority and hospital care. Results from the logistic regression models are shown in Table 4. Furthermore, due to the inverse association between ED-LOS and mortality between patients triaged with priority 1 and patients triaged with priority 2–4, data were grouped into two groups, patients with triage priority 1 (high priority) and patients with triage priority 2–4 (lower priority).

Our data show that 7-day mortality was negatively associated with ED-LOS in patients with triage priority 1 and admitted to in-hospital care, OR 0.92 (0.90–0.94 CI 95%). A similar observation was made for triage priority group 2–4, however, not significant, OR 0.98 (0.97–1.00 CI 95%). A comparable observation was made for the outcome 30-day mortality. In contrast, in patients not admitted in-hospital, we observed a positive association between ED-LOS and 7- and 30-day mortality, independently of triage priority (Table 4). When looking at age categories, patients in highest triage priority group showed lower mortality risk with increased ED-LOS for both 7- and 30-day mortality, while patients in triage groups 2–4 showed higher mortality risk with increased ED-LOS for 30-day mortality (Table 5).

## Discussion

In this study we observed a positive association between ED-LOS 7- and 30-day mortality rate in patients with lower medical urgency and patients not admitted to in-hospital care. In contrast, this was not observed in patients with high medical urgency and in patients admitted to in-hospital care. The clinical impact of these findings remains to be elucidated.

The effects of long ED-LOS and ED crowding on non-favourable outcome have been well studied [1], however, very sparsely in the Swedish settings [10]. Changes in the vital signs, directing the patient to high triage priority, are in RETTS-A closely related to 1-day mortality [12]. Therefore, is anticipated that patients with very urgent symptoms and deranged vital parameters have a high mortality [13], in the present study reflected by a high mortality in patients with triage priority 1. It has been shown that long ED-LOS is harmful for patients with high degree of medical urgency (high triage priority), as for example for patients with non-ST-segment-elevation myocardial infarction [14] or for patients with sepsis [15]. We were not able to find any association between increased mortality and increased ED-LOS in patient with triage priority 1, instead, a negative association was observed. The most plausible explanation for this is that acute ill patient quickly pass/bypass the ED for further care. Mortality in these patients is high and time to definitive care is crucial and it is expected that some of these patients use medical “fast tracks” for example for patients with neurological deficits or ST-elevation myocardial infarctions [16]. In addition, some acute ill patients with high medical urgency will be treated immediately at the ED and not bypass the ED. Here the outcome and ED-LOS will be dependent on the ability to stabilise the patients and the decision for further admission or not.

In contrast to high priority patients, mortality in patients with lower triage was positively associated with ED-LOS. One explanation for this finding could be an effect of under-triage, where patients with medical urgency remain undetected by the triage tool. It is well described that patients with non-specific symptoms and low clinical urgency often have increased hospitalisation, increased ED-LOS, increased mortality and more often are of advanced age and frail [10, 17, 18]. A French study on stay at ED plus an observation unit (EDOU-LOS), found that older age was associated with longer EDOU-LOS [8]. Older patients do have a more complex situation, and both decision-making and gaining access to diagnostic tools may take longer time. Different triage tools are often validated against proxy outcomes of ED performance such re-admission rate or mortality. Importantly, however, the main aim for triage, including the use of RETTS-A, is to identify clinical urgency and not to predict 7- or 30-day mortality, thus problematizing the difficulties to study effects of under-triage [19]. In accordance with our findings, Berg et al. [10] also observed a positive association between increased ED-LOS and short-term mortality in ED patients with low medical priority (RETTS-A levels 3–5) and not admitted to in-hospital care. In addition, an Australian study found similar results [20]. In contrast, a Canadian study found that patients who left without being examined were not at higher risk of short-term adverse events [21]. In addition, we observed no association between long ED-LOS and short-term mortality in patients admitted to in-hospital care, regardless of triage priority suggesting a favourable effect of in-hospital care. Importantly, a low availability of in-hospital beds (in-hospital occupancy) has been suggested to be associated to a lower probability for patient admittance to in-hospital care [22]. We cannot exclude that in-hospital occupancy could contribute to our findings, and further studies are warranted to study this in more detail.

Studies to ameliorate the prognosis of long ED-LOS patients with lower medical urgency have been performed. One way could be to create a “fast track” to “Day-Hospital Services” [23]. An article on the ED overcrowding in an international perspective, concluded that the causes of it is a “complex network of interwoven processes”, and that ED overcrowding could not be solved in an easy manner [24]. ED boarding is pointed out as one of the main factors, but it is noted that more overall actions from the emergency physicians and hospitals are needed, and that future works are needed to evaluate different interventions as a base of evidence-based guidelines. A simple rule was proposed, i.e. that 90% of the ED patients should leave the ED within 6–8 h, but also keeping in mind that targets could not overrule clinical judgements.

ED-LOS is regarded as a key ED performance indicator in Sweden and is followed up annually by the National Board of Health and Welfare. A major trend, for the whole Swedish population, has been that ED-LOS is longer for elderly patients’ ≥ 80 years of age compared to patients 19–79 years[25]. In this study, elderly patients had the longest ED-LOS which is in line with the national annual survey [25]. Elderly patients are especially exposed to the negative effects of ED crowing due to their higher comorbidity, lower physiological reserves and diffuse symptoms of medical urgency leading to under-triage [26]. In line with the literature, our data indicated a small increase in 30-day mortality in elderly patients with low clinical urgency and long ED-LOS. Unfortunately, our data do not allow us to study this further.

## Strengths and limitations

One strength in our study is the large cohort (approximately 600,000 visits during a 5-year period) and thus generalizable to the population at large. A major limitation was that we did not have access to all relevant underlying diagnoses and comorbidities of the included patients. Thus, we had not the possibility to adjust results for frailty scores. We have tried to compensate for this by adding several “proxies” for patient comorbidity and short-term mortality in the regression models. These proxies included mainly the patients final ED diagnose and the patients’ chief complaint as well as the age of the patient but also if the patient received prehospital care or not. Patients’ ED diagnoses are a well-accepted predictor of short-term mortality. The patients primary ED diagnoses and the patients ED chief complaint as well as the combination of chief complaint have been closely associated to patient outcome such as hospitalisation and mortality [27, 28]. Age has also been a powerful predictor of short-term mortality in the ED [12]. Underlying diagnoses and comorbidities are not used in RETTS-A and should therefore have less impact on ED-LOS compared to patients’ chief complaint or triage level assumed to be of more relevance to the clinical milieu at the ED than comorbidity. Another limitation is that this is an observational study. In the current study, we included all patients seeking care at the ED at two large hospitals in our analyses. Another limitation was that we did not had access to the degree of crowding in our analyses. Just recently, one of the first studies exploring the effect of crowing on mortality in the Swedish setting was published. Here the authors found that mortality increases in patients with low triage priority when ED occupancy increases [10]. In addition, to assure a rapid care and a low ED-LOS for critically ill patients, different fast tracks have been developed to quickly bypass the crowded ED and to reduce mortality [29]. Such fast tracks are recommended for example for patients with acute chest pain or stroke [16] and were during the study time operating in both university hospitals. Finally, we have not studied the special effects of long ED-LOS on specific vulnerable patient groups such as frail patients.

## Clinical implications

The observed differences in this study are small and suggest no causality between increased ED-LOS and short-term mortality in general. Nevertheless, our results confirm recently published findings that in the Swedish setting, prolonged ED-LOS in patients with lower medical priority and dismissed home from the ED, is associated with increased short-term mortality. The new finding here is that this observation is not seen in patients admitted to in-hospital care. Strategies for a swift change from in-hospital care to primary care already exist in the Swedish care system for patients planned to be dismissed from the hospital ward, similar solutions should be operable also for patients leaving the ED. In general, ED-LOS for some patients especially with lower priority seem unacceptably long.

## Conclusion

Our data suggest that prolonged ED-LOS could be harmful to patients not admitted to in-hospital care and with low triage priority. The clinical impact of these findings remains to be explored.

## Data Availability

The datasets used and/or analysed during the current study are available from the corresponding author on reasonable request.

## Abbreviations

ED: Emergency department
ED-LOS: Emergency department length of stay
RETTS-A: Rapid Emergency Triage and Treatment system

## Declarations

### Patient and public involvement statement

Patients or the public WERE NOT involved in the design, or conduct, or reporting, or dissemination plans of our research.
